# Triple detection of the tumor proteins p63, Ki-67 and Alpha-methylacyl-CoA racemase in the pathological diagnosis of prostate cancer

**DOI:** 10.5937/jomb0-60143

**Published:** 2026-04-15

**Authors:** Ming Niu, Danbo Zhao, Ruixue He, Ling Qin, Zhikai Li, Side Zhang, Lijun Chen

**Affiliations:** 1 Tianshui Integrated Traditional Chinese and Western Medicine Hospital, Department of Urology, Tianshui City, China; 2 Ezhou Central Hospital, Department of Oncology, Ezhou City, China; 3 Air Force Hospital of Western Theatre Command, PLA, Department of Urology, Chengdu City, China; 4 Sun Yat-sen University First Affiliated Hospital, Department of Urology, Guangzhou City, China; 5 Peking University Shenzhen Hospital, Department of Urology, Shenzhen City, China

**Keywords:** prostate tumour, immunohistochemistry, Alpha-methylacyl-CoA racemase (AMACR), p63 protein, Ki-67 antigen, tumor prostate, imunohistohemija, alfa-metilacil-CoA racemaza (AMACR), p63 protein, Ki-67 antigen

## Abstract

**Background:**

To explore the value and significance of tripleimmunohistochemical detection of alpha-methylacyl-CoAracemase (AMACR), p63 and Ki-67 in the pathologicaldiagnosis of prostate cancer.

**Methods:**

A total of 156 paraffin-archived prostate biopsysamples collected from our hospital from June 2023 toAugust 2024 were selected as research materials. Theexpression levels of AMACR, p63 and Ki-67 in puncturesamples from 48 patients with prostate cancer, 32 patientswith high-grade prostate intraepithelial neoplasia, 26patients with low-grade prostate intraepithelial neoplasiaand 50 patients with benign prostatic hyperplasia weredetected via immunohistochemistry.

**Results:**

There were statistically significant differences in thepositive expression rates of AMACR, p63 and Ki-67 amongthe different groups of samples. The positive expression rateof AMACR in the puncture samples of the prostate cancergroup was 100%, with a high expression rate of 81.25%. The negative expression rate of p63 was 97.92%, whichwas significantly greater than that of the other three groups.The positive expression rate of Ki-67 was 81.25%, and thehigh expression rate was 54.17%. The AMACR is related tothe long diameter of the tumour, TNM stage, degree of differentiation and Gleason score in patients with prostate cancer. The rates of high AMACR in patients with tumours withlong diameters S1.5 cm, stage II-III, moderate to highly differentiated, and Gleason scores ranging from 8-10 weresignificantly higher than those in patients with long diameters &lt;1.5 cm, stage I. The expression of Ki-67 is related tothe long diameter of the tumour, degree of differentiation,degree of lymph node metastasis and Gleason score inpatients with prostate cancer. The rates of high expressionof Ki-67 in patients with a long tumor diameter of S1.5 cm,moderate and high differentiation, and lymph node metastasis and a Gleason score of 8-10 were significantly greaterthan those in patients with a long tumor diameter of &lt;1.5cm, poor differentiation, no lymph node metastasis, and aGleason score of 2-7. p63 was not related to the clinical orpathological characteristics of patients with prostate cancer(all P&gt;0.05). The sensitivity and negative predictive value ofthe combined diagnosis of AMACR-positive/p63-negative/Ki-67-positive prostate cancer were both 100.00%,and the specificity was 81.82%.

**Conclusions:**

AMACR, p63 and Ki-67 in prostate biopsysamples can be used as promising biomarkers for the diagnosis or exclusion of prostate cancer. Combined detectioncan improve the accuracy of prostate cancer diagnosis.

## Abstract (sr)

**Uvod:**

Cilj je bio ispitivanje vrednosti i značaja trostrukeimunohistohemijske detekcije alfa-metilacil-CoA racemaze(AMACR), p63 i Ki-67 u patološkoj dijagnozi karcinomaprostate.

**Metode:**

Za istraživanje je korišćeno ukupno 156 parafinskih uzoraka biopsije prostate, prikupljenih u našoj bolniciod juna 2023. do avgusta 2024. godine. EkspresijaAMACR, p63 i Ki-67 analizirana je imunohistohemijom upunkcionim uzorcima od 48 pacijenata sa karcinomomprostate, 32 pacijenta sa intraepitelijalnom neoplazijomprostate visokog stepena, 26 pacijenata sa intraepitelijalnom neoplazijom prostate niskog stepena i 50 pacijenatasa benignom hiperplazijom prostate

**Rezultati:**

Postojale su statistički značajne razlike u stopamapozitivne ekspresije AMACR, p63 i Ki-67 među različitimgrupama uzoraka. Stopu pozitivne ekspresije AMACR upunkcionim uzorcima iz grupe karcinoma prostate iznosilaje 100%, sa visokim nivoom ekspresije od 81,25% . Negativna ekspresija p63 iznosila je 97,92% , sto je značajno visenego u ostalim grupama. Stopa pozitivne ekspresije Ki-67bila je 81,25% , dok je visok nivo ekspresije iznosio 54,17%.Ekspresija AMACR bila je povezana sa najvecim prečnikomtumora, TNM stadijumom, stepenom diferencijacije iGleason skorom kod pacijenata sa karcinomom prostate.Stope visoke ekspresije AMACR kod pacijenata sa tumorima čiji je najveci prečnik S1,5 cm, stadijum II-III, umerenedo visoke diferecijacije, Gleason skor 8-10, bile su značajno vise nego kod pacijenata sa prečnikom &lt; 1,5 cm istadijumom I. Ekspresija Ki-67 bila je povezana sa najvecimprečnikom tumora, stepenom diferencijacije, prisustvommetastaza u limfnim čvorovima i Gleason skorom. Stopevisoke ekspresije Ki-67 kod pacijenata sa prečnikom tumora S1,5 cm, umereno i visoko diferentisanim tumorima, sametastazama u limfnim čvorovima i Gleason skorom 8-10bile su značajno vise nego kod pacijenata sa prečnikom&lt; 1,5 cm, niskim stepenom diferencijacije, bez metastaza iGleason skorom 2-7. Ekspresija p63 nije bila povezana sakliničkim ili patoloskim karakteristikama pacijenata (sveP&gt; 0,05). Osetljivost i negativna prediktivna vrednost kombinovane dijagnoze AMACR-pozitivnog/p63-negativnog/Ki-67-pozitivnog karcinoma prostate bile su 100%, dok jespecifičnost iznosila 81,82%.

**Zaključak:**

AMACR, p63 i Ki-67 u uzorcima biopsijeprostate se mogu koristiti kao perspektivni biomarkeri zadijagnozu ili isključenje karcinoma prostate. Kombinovanadetekcija može značajno poboljsati tačnost dijagnoze.

## Introduction

Prostate cancer has become the sixth most common malignant tumour among men in China.Moreover, with the intensification of the ageing process and the advancement of screening technologies, the incidence and mortality rates of prostate cancer in China have been increasing annually [1][2]. At present, in clinical practice, prostate cancer is screened mainly through serum prostate-specific antigens. However, this indicator has low sensitivity and specificity, and there is a relatively wide grey area [3][4]. Although pathological examination of prostate biopsy samples is the gold standard for the diagnosis of prostate cancer, the number of puncture samples is limited, the morphological differences between benign and malignant lesions are minor, and missed diagnoses often occur under light microscopy. Therefore, it is necessary to search for new specific tumour markers to improve the detection rate of prostate cancer and the accuracy of differentiating between benign and malignant lesions [5]. Studies have shown that α-methylacyl-CoA racemase (AMACR) is positively expressed in the cytoplasm of renal cancer, gastric cancer and prostate cancer cells and has good sensitivity and specificity for the diagnosis of these cancers [6][7]. p63 is a widely used basal cell-specific marker [8], and Ki-67 is a commonly used proliferation marker in tumour diagnosis [9]. Clinical studies have shown that the combined detection of multiple indicators helps improve the detection rate and accuracy of specific diseases [10].

AMACR, p63 and Ki-67 in biopsy tissue samples of prostate lesions of different grades were evaluated, and their correlations with the baseline data and pathological characteristics of patients were explored.

## Materials and methods

### Research subjects

A total of 156 paraffin-archival prostate biopsy samples collected from our hospital from June 2023 to August 2024 were selected as research materials, including 48 prostate cancer samples, 32 high-grade prostate intraepithelial neoplasia (HGPIN) samples, 26 low-grade prostate intraepithelial neoplasia (LGPIN) samples, and 50 benign prostatic hyperplasia samples. Patients with prostate cancer were aged 43-76 (61.8±7.0) years; their body mass index (BMI) was 21-32.5 (25.49±3.19) kg/m; patients with HGPIN were aged 42-75 (61.0±7.6) years; their BMI was 20.2-31.9 (25.18±3.65) kg/m; patients with LGPIN were aged 42-77 (61.64±6.52) years; their BMI was 20.5-30.5 (24.47±3.02) kg/m; patients with benign prostatic hyperplasia were aged 42-73 (60.53±5.95) years; and their BMI was 20.4-31.3 (24.92±3.83) kg/m. This study has been approved by the Ethics Committee for Human Medical Research (HKYS-2025-A0159).

### Inclusion and exclusion criteria

The inclusion criteria were as follows: (1) no radiotherapy, chemotherapy, targeted therapy or immunotherapy was performed before enrollment; and (2) radical prostatectomy, prostate biopsy, or first diagnosis of prostate cancer, prostate intraepithelial neoplasia or benign prostatic hyperplasia through imaging examinations.

The exclusion criteria were as follows: (1) had other malignant tumours; (2) had recurrent prostate cancer; (3) had incomplete or missing clinical data; and (4) had bone metastasis.

### Reagents and equipment

Murine antihuman AMACR monoclonal antibody (ab268062), murine antihuman p63 monoclonal antibody (ab32389), and murine antihuman Ki67 monoclonal antibody (ab245113) were purchased from Abcam Company in the United States. The immunohistochemical kit, DAB staining kit and mounting adhesive were purchased from Fuzhou Maixin Biotechnology Development Co., Ltd. A BX41 optical microscope was purchased from Olympus Corporation of Japan.

The experiment used paraffin-embedded tissue samples (4 pm thickness) from prostate biopsy and radical resection, approved by the ethics committee. The slices were baked at 60 for 1 hour and then dewaxed with xylene (Sinopharm Group, X8200) and hydrated with gradient ethanol (100%, 95%, 80%; Sinopharm Group) in sequence. Rinse three times with PBS buffer (Solarbio, P1020). Treat with EDTA antigen remediation solution (pH 9.0, Biyun Tian, P0085) in a high-pressure remediation pot (Eppende, E1-1002) at 121°C for 15 minutes. After natural cooling, add 3% H O (Maixin Bio, KIT-5010) and block endogenous peroxidase at room temperature for 15 minutes. After PBS rinsing, add 5% BSA blocking solution (Sigma, A8020) and incubate at room temperature for 30 minutes. Staining was performed using a fully automatic immunohistochemical instrument (Roche BenchMark ULTRA, item No. 06546519001). Each batch of the experiment was set with a positive control (known prostate cancer tissue) and a negative control (PBS instead of the primary antibody). Image acquisition was performed using a digital pathological scanner (Aperio AT2, Leica, product number GT450DX).

### Data collection

The baseline data of the patients (age, BMI, underlying disease, prostate volume, etc.) and pathological characteristics (long diameter of the tumour, clinical stage, degree of differentiation, lymph node metastasis, Gleason pathological grade score) were collected.

### Immunohistochemical detection

The paraffin blocks of prostate puncture samples were cut into thin slices with a thickness of 4 μm. AMACR, p63 and Ki-67 were detected via streptomycin antibiotic protein peroxidase immunohistochemical (S-P) staining. Prostate biopsy samples that were positive for AMACR, p63 and Ki-67 were used as positive controls.

p63 (anti-mouse HRP polymer): DAKO (Agilent), M7247; Ki-67 (anti-rabbit AP polymer): Abcam, ab16667; AMACR (anti-mouse HRP polymer): DAKO (Agilent), M3616.

### Judgment of the immunohistochemical results

The appearance of fine particles ranging from light yellow to brownish yellow at specific locations in the cells was regarded as positive. Among them, AMACR is located in the cytoplasm, p63 is located in the basal cell nucleus, and Ki-67 is located in the cell nucleus. For a positive cell percentage score, 10% is worth zero points, >10% to 25% is worth one, >25% to 50% is worth two, >50% to 75% is worth three, and >75% is worth four. (2) Staining intensity score: 0 points for either hazy or no colour development. Each indicator is divided into two halves with the median expression level as the boundary.

### Statistical analysis

SPSS 25.0 software was used to analyse the data collected for this investigation. The χ^2^ test was used to compare groups, and count statistics are expressed as the number of cases (percentages). Pearson analysis was used to evaluate the correlation of the expression levels of AMACR, p63 and Ki-67 in prostate cancer biopsy samples. A P value <0.05 was considered to indicate statistical significance.

## Results

### Comparison of the expression of AMACR, p63 and Ki-67 in different puncture samples

The positive expression rate of AMACR in the puncture samples of the prostate cancer group was 100%, with a high expression rate of 81.25%. The negative expression rate of p63 was 97.92%, which was significantly greater than that of the other three groups. The positive expression rate of Ki-67 was 81.25%, among which the high expression rate was 54.17% (Table 1).

**Table 1:** Comparison of the expression of AMACR, p63 and Ki-67 in different puncture samples (%).

Group	Number<br>of cases	AMACR			p63			Ki-67		
		Negative	Low	High	Negative	Low	High	Negative	Low	High
Prostate<br>cancer	48	0	9 (18.75)	39 (81.25)	47 (97.92)	1 (2.08)	0	9 (18.75)	13 (27.08)	26 (54.17)
HGPIN<br>group	32	0	14 (43.75)	18 (56.25)	2(6.25)	17 (53.12)	13 (40.63)	19 (59.38)	11 (34.37)	2 (6.25)
LGPIN<br>group	26	4 (15.39)	17 (65.38)	5 (19.23)	0	6 (23.08)	20 (76.92)	20 (76.92)	6 (23.08)	0
Benign<br>Prostatic	50	49 (98.00)	1 (2.00)	0	0	2 (4.00)	48 (96.00)	47 (94.00)	3 (6.00)	0
x^2^ value	-	119.163	-	-	126.431	-	-	72.030	-	-

### Relationships between AMACR, p63 and Ki-67 in prostate cancer puncture samples and clinico-pathological characteristics

The expression of AMACR is related to the long diameter of the tumour, TNM stage, degree of differentiation and Gleason score in patients with prostate cancer. The rates of high AMACR in patients with tumors with long diameters 21.5 cm, stage II-III, moderate to highly differentiated, and Gleason scores ranging from 8-10 were significantly higher than those in patients with long diameters <1.5 cm, stage I, poorly differentiated, and Gleason scores ranging from 2-7, respectively (all P<0.05). The expression of Ki-67 is related to the long diameter of the tumour, degree of differentiation, degree of lymph node metastasis and Gleason score in patients with prostate cancer. The rates of high expression of Ki-67 in patients with a long tumor diameter of ≥1.5 cm, moderate and high differentiation, and lymph node metastasis and a Gleason score of 8-10 were significantly greater than those in patients with a long tumor diameter of <1.5 cm, poor differentiation, no lymph node metastasis, and a Gleason score of 2-7 (all P<0.05). The expression of p63 in patients with prostate cancer was not related to clinical or pathological characteristics (all P>0.05), as shown in Table 2.

**Table 2:** Relationship between the expressions of AMACR, p63 and KI-67 and clinicopathological characteristics in 48 prostate cancer biopsy samples (%).

Item	Number<br>of cases	AMACR				p63				Ki-67			
		High	Low	x^2^ value	P value	Negative	High	x^2^ value	P value	High	Low	x^2^ value	P value
Age	-	-	-	0.697	0.404	-	-	0.864	0.353	-	-	1.467	0.226
≥60 age	22	19<br>(86.36)	3<br>(13.64)	-	-	22<br>(100.00)	0	-	-	14<br>(63.64)	8<br>(36.36)	-	-
<60 age	26	20<br>(76.92)	6<br>(23.08)	-	-	25<br>(96.15)	1<br>(3.85)	-	-	12<br>(46.15)	14<br>(53.85)	-	-
Hypertension	-	-	-	1.691	0.193	-	-	2.750	0.097	-	-	0.390	0.532
Yes	13	9<br>(69.23)	4<br>(30.77)	-	-	12<br>(92.31)	1<br>(7.69)	-	-	8<br>(61.54)	5<br>(38.46)	-	-
No	35	30<br>(85.71)	5<br>(14.29)	-	-	35<br>(100.00)	0	-	-	18<br>(51.43)	17<br>(48.57)	-	-
Diabetes	-	-	-	1.049	0.306	-	-	3.881	0.069	-	-	1.275	0.259
Yes	10	7<br>(70.00)	3<br>(30.00)	-	-	9<br>(90.00)	1<br>(10.00)	-	-	7<br>(70.00)	3<br>(30.00)	-	-
No	38	32<br>(84.21)	6<br>(15.79)	-	-	38<br>(100.00)	0	-	-	19<br>(50.00)	19<br>(50.00)	-	-
Coronary heart<br>disease	-	-	-	1.654	0.198	-	-	0.119	0.730	-	-	0.076	0.782
Yes	5	3<br>(60.00)	2<br>(40.00)	-	-	5<br>(100.00)	0	-	-	3<br>(60.00)	2<br>(40.00)	-	-
No	43	36<br>(83.72)	7<br>(16.28)	-	-	42<br>(97.67)	1<br>(2.33)	-	-	23<br>(53.49)	20<br>(46.51)	-	-
Prostate volume	-	-	-	0.259	0.611	-	-	0.940	0.332	-	-	0.071	0.790
≥50 mL	23	18<br>(78.26)	5<br>(21.74)	-	-	23<br>(100.00)	0	-	-	12<br>(52.17)	11<br>(47.83)	-	-
<50 mL	25	21<br>(84.00)	4<br>(16.00)	-	-	24<br>(96.00)	1<br>(4.00)	-	-	14<br>(56.00)	11<br>(44.00)	-	-
Long diameter<br>of the tumour	-	-	-	6.757	0.009	-	-	0.669	0.413	-	-	15.791	<0.001
≥1.5 cm	19	12<br>(63.16)	7<br>(36.84)	-	-	19<br>(100.00)	0	-	-	17<br>(89.47)	2<br>(10.53)	-	-
<1.5 cm	29	27<br>(93.10)	2<br>(6.90)	-	-	28<br>(96.55)	1<br>(3.45)	-	-	9<br>(31.03)	20<br>(68.97)	-	-
TNM Installment	-	-	-	4.729	0.030	-	-	0.560	0.454	-	-	1.789	0.181
Stage I	17	11<br>(64.71)	6<br>(35.29)	-	-	17<br>(100.00)	0	-	-	7<br>(41.18)	10<br>(58.82)	-	-
Stage II - III	31	28<br>(90.32)	3<br>(9.68)	-	-	30<br>(96.77)	1<br>(3.23)	-	-	19<br>(61.29)	12<br>(38.71)	-	-
Degree of<br>differentiation	-	-	-	9.156	0.010	-	-	1.862	0.394	-	-	22.564	<0.001
Low<br>differentiation	17	10<br>(58.82)	7<br>(41.18)	-	-	16<br>(94.12)	1<br>(5.88)	-	-	2<br>(11.76)	15<br>(88.24)	-	-
Moderate<br>differentiation	20	18<br>(90.00)	2<br>(10.00)	-	-	20<br>(100.00)	0	-	-	13<br>(65.00)	7<br>(35.00)	-	-
High<br>differentiation	11	11<br>(100.00)	0	-	-	11<br>(100.00)	0	-	-	11<br>(100.00)	0	-	-
Lymph node<br>metastasis	-	-	-	1.396	0.237	-	-	0.669	0.413	-	-	20.850	<0.001
Yes	19	17<br>(89.47)	2<br>(10.53)	-	-	19<br>(100.00)	0	-	-	18<br>(94.74)	1<br>(5.26)	-	-
No	29	22<br>(75.86)	7<br>(24.14)	-	-	28<br>(96.55)	1<br>(3.45)	-	-	8<br>(27.59)	21<br>(82.41)	-	-
Gleason Score	-	-	-	10.843	0.004	-	-	3.435	0.179	-	-	13.739	0.001
G1(2~4points)	11	6<br>(54.55)	5<br>(45.45)	-	-	10<br>(90.91)	1<br>(9.09)	-	-	1<br>(9.09)	10<br>(90.91)	-	-
G2(5~7points)	15	11<br>(73.33)	4<br>(26.67)	-	-	15<br>(100.00)	0	-	-	8<br>(53.33)	7<br>(46.67)	-	-
G3 (8~10points)	22	22<br>(100.00)	0	-	-	22<br>(100.00)	0	-	-	17<br>(77.27)	5<br>(22.73)	-	-

### Analysis of the efficacy of AMACR and p63 combined with Ki-67 in the diagnosis of prostate cancer in puncture samples

The efficacy and parameters of positive AMACR, negative p63, positive Ki-67 and positive AMACR/negative p63/positive Ki-67 in the diagnosis of prostate cancer in puncture samples are detailed in Table 3 and Table 4. Positive AMACR/negative p63/positive Ki-67 as diagnostic criteria for prostate cancer can achieve the best sensitivity and negative predictive value, both of which are 100%.

**Table 3:** Diagnosis of prostate cancer by puncture pathology and AMACR, p63, and KI-67.

Pathological<br>diagnosis	AMACR		p63		Ki-67		Tota
	+	-	+	-	+	-	
Prostate cancer	39	9	1	47	26	22	48
Nonprostate cancer	23	85	81	27	2	106	108
**Total**	**62**	**94**	**82**	**74**	**28**	**128**	**156**

**Table 4:** Efficacy paarmeters of AMACR, p63 and KI-67 in the diagnosis of prostate cancer (%).

Indicator	Sensitivity	Specificity	Positive	Negative
AMACR	62.90	90.43	81.25	78.70
p63	63.51	98.78	97.92	75.00
Ki-67	92.86	82.81	54.17	98.15
AMACR<br>positive/p63<br>negative/Ki-67<br>positive	100.00	81.82	50.00	100.00

## Discussion

Prostate cancer is a primary malignant tumour of the male reproductive system. Pathological examination after prostate biopsy sampling is the »gold standard« for the diagnosis of prostate cancer. However, owing to the complex tissue structure of prostate cancer, simple microscopic morphological examination sometimes has difficulty making a precise diagnosis of precancerous lesions and benign lesions similar to those of cancer. Therefore, some immune markers are needed to provide an auxiliary diagnostic basis [11][12].

In this study, immunohistochemistry was used to detect markers such as AMACR, p63 and Ki-67 in prostate biopsy samples from patients with different prostate lesions, such as prostate cancer, HGPIN, LGPIN and benign prostatic hyperplasia. The results revealed positive expression rates and high expression rates of AMACR and Ki-67 in the prostate cancer group. The p504s gene encodes AMACR, and its main function is to catalyse the β-oxidation of branched-chain fatty acids and their derivatives. The positive expression rate of AMACR is very high in prostate cancer tissues. Some studies have shown that the positive expression rate is as high as 94%-100%, whereas no positive expression is found in benign lesions [13][14][15]. There was 1 case of positive expression in the benign prostatic hyperplasia group, and it was expressed at a low level. The positive expression rates of AMACR in the HGPIN and LGPIN groups were also as high as 100.00% and 84.62%, respectively, which was consistent with previous research conclusions. Therefore, AMACR can be used as a highly sensitive and specific positive marker for prostate cancer and precancerous lesions. The occurrence of prostate cancer is related to long-term high levels of cytoplasmic branched-chain fatty acids [16][17]. Phytate acid is a kind of branched-chain fatty acid that is relatively abundant in red meat and dairy products and is mainly degraded by AMACR. When the intake level is high, the expression of AMACR is upregulated to degrade phytate acid [18]. The results of this study also revealed that the expression of AMACR in prostate cancer biopsy samples was related to the long diameter of the tumour, TNM stage, degree of differentiation and Gleason score. Patients with a long diameter of the tumour ≥1.5 cm, stage II-III disease, moderate to highly differentiated disease, and a Gleason score of 8-10 had a higher rate of high expression of AMACR.

p63 is a member of the p53 family and is specifically expressed in the basal cell nuclei of the prostate. In normal prostate tissues, basal cells have good continuity, and p63 is positively expressed. However, in malignant prostate lesion tissues, basal cells are absent, and p63 is not expressed or expressed at a low level [19][20]. In this study, the negative expression rate of p63 in the puncture samples of the prostate cancer group was 97.92%, which was significantly greater than that of the other three groups. Therefore, p63 can be used as an indicator of the status of glandular basal cells and can also be used as an auxiliary indicator for the exclusion of prostate cancer.

Ki-67 is a cell proliferation marker that can detect the proportion of cells in the cell cycle and is used to evaluate the malignancy, grade and prognosis of tumours [21][22][23][24]. High Ki-67 expression is usually associated with high proliferative activity of tumour cells and a poor prognosis [25][26][27]. In this study, the degree of differentiation, the degree of lymph node metastasis, and the Gleason score were measured. The high expression rate of Ki-67 was greater in patients with tumours with long diameters (≥1.5 cm), moderate to high differentiation, lymph node metastasis, and Gleason scores ranging from 8 to 10. Relevant studies [28][29][30] have shown that high expression of Ki-67 is closely related to poor prognosis in prostate cancer patients.

This study simultaneously analysed the efficacy of AMACR-positive, p63-negative, and Ki-67-positive puncture samples, as well as AMACR-positive/p63-negative/Ki-67-positive samples, in diagnosing prostate cancer. The results indicated that AMACR-positive/p63-negative/Ki-67-positive status, as the diagnostic criterion for prostate cancer, could achieve the best sensitivity and negative predictive value. All are 100%, which is superior to individual detection.

## Conclusion

AMACR, p63 and Ki-67 in prostate biopsy samples can be used as promising biomarkers for the diagnosis or exclusion of prostate cancer, and combined detection can improve the accuracy of prostate cancer diagnosis.

## Dodatak

### Authors contribution

Ming Niu and Danbo Zhao contributed equally to this work as first authors.

### Conflict of interest statement

All the authors declare that they have no conflict of interest in this work.

## References

[1] Melnychuk M R (2023). Diagnostic Utility Of Immunohisto-chemical Expression Of Ki!67, R63 And Amacr In Prostate Intraepithelial Neoplasia. Wiad Lek.

[2] Rathod S, Jaiswal D, Bindu R (2019;Aug 28). Diagnostic utility of triple antibody (AMACR, HMWCK and P63) stain in prostate neoplasm. J Family Med Prim Care.

[3] Ramatlho P, Rugemalila M, Tawe L, Pain D, Choga O, Ndlovu A, Koobotse M, Lal P, Rebbeck T, Paganotti G, Narasimhamurthy M, Kyokunda L (2025;Jan 22). The Role of Immunohistochemistry for AMACR/p504s and p63 in Distinguishing Prostate Cancer from Benign Prostate Tissue Samples in Botswana. Res Rep Urol.

[4] Boran C, Kandirali E, Yilmaz F, Serin E, Akyol M (2011;Nov-Dec). Reliability of the 34βE12, keratin 5/6, p63, bcl-2, and AMACR in the diagnosis of prostate carcinoma. Urol Oncol.

[5] Ng V W L, Koh M, Tan S Y, Tan P H (2007;Feb). Is Triple Immunostaining With 34βE12, p63, and Racemase in Prostate Cancer Advantageous? A Tissue Microarray Study. Am J Clin Pathol.

[6] Yu T, Zhu S X, Zheng S, Chen S R (2007;Mar;). Detection of AMACR (R504S), R63 and 34betaE12 cocktail in the early diagnosis of prostate cancer (Chinese). Zhonghua Nan Ke Xue.

[7] Herawi M, Epstein J I (2007;Jun). Immunohistochemical Antibody Cocktail Staining (p63/HMWCK/AMACR) of Ductal Adenocarcinoma and Gleason Pattern 4 Cribriform and Noncribriform Acinar Adenocarcinomas of the Prostate. Am J Surg Pathol.

[8] Molinié V, Vieillefond A, Michiels J (2008;Oct). Évaluation des marqueurs p63 et p504s dans le diagnostic du cancer de prostate. Ann Pathol.

[9] Čamdžić N, Kuskunović-Vlahovljak S, Dorić M, Radović S, Lazović S E, Babić M (2020;Feb 1). Serum total prostate-specific antigen (tPSA): Correlation with diagnosis and grading of prostate cancer in core needle biopsy. Med Glas (Zenica).

[10] Mcdaniel A S, Chinnaiyan A M, Siddiqui J, McKenney J K, Mehra R (2014;Dec). Immunohistochemical Staining Characteristics of Nephrogenic Adenoma Using the PIN-4 Cocktail (p63, AMACR, and CK903) and GATA-3. Am J Surg Pathol.

[11] Hameed O, Humphrey P A (2005;Nov). p63/AMACR Antibody Cocktail Restaining of Prostate Needle Biopsy Tissues After Transfer to Charged Slides: A Viable Approach in the Diagnosis of Small Atypical Foci That Are Lost on Block Sectioning. Am J Clin Pathol.

[12] Pierconti F, Rossi E D, Martini M, Sacco E, Bassi P F, Larocca L M (2019;Apr). 34BetaE12 and Alfa-Methylacyl Coenzyme A Racemase (AMACR) Antibodies Better Than p63 Antibody Distinguish Normal and Neoplastic Glands in Prostatic Tissue With Thermal Artifacts. Appl Immunohistochem Mol Morphol.

[13] Wu L, Zheng Y, Liu J, Luo R, Wu D, Xu P, Wu D, Li X (2021;Apr 20). Comprehensive evaluation of the efficacy and safety of LPV/r drugs in the treatment of SARS and MERS to provide potential treatment options for COVID-19. Aging (Albany NY).

[14] Browne T, Hirsch M S, Brodsky G, Welch W R, Loda M F, Rubin M A (2004;Dec). Prospective evaluation of AMACR (P504S) and basal cell markers in the assessment of routine prostate needle biopsy specimens. Hum Pathol.

[15] Jiang Z, Li C, Fischer A, Dresser K, Woda B A (2005;Feb). Using an AMACR (P504S)/34βE12/p63 Cocktail for the Detection of Small Focal Prostate Carcinoma in Needle Biopsy Specimens. Am J Clin Pathol.

[16] Wu L, Zhong Y, Wu D, Xu P, Ruan X, Yan J, Liu J, Li X (2022;Sep 10). Immunomodulatory Factor TIM3 of Cytolytic Active Genes Affected the Survival and Prognosis of Lung Adenocarcinoma Patients by Multi-Omics Analysis. Biomedicines.

[17] Manu V, Singh V, Malik A, Dutta V, Mani N S, Patrikar S (2014;Jul-Sep). Diagnostic utility of p63 and α-methyl acyl Co A racemase in resolving suspicious foci in prostatic needle biopsy and transurethral resection of prostate specimens. J Cancer Res Ther.

[18] Liu Y N, Jiang Z M, Wang X Y, Zhang H Z, Chen J Q, Huang J, Yang Q H (2006;Jul). The value of using an AMACR/34betaE12/p63 cocktail double staining for diagnosis of prostate carcinoma and precarcinomatous lesions (Chinese). Zhonghua Bing Li Xue Za Zhi.

[19] Krzyzanowska A, Lippolis G, Helczynski L, Anand A, Peltola M, Pettersson K, Lilja H, Bjartell A (2016;May). Quantitative Time-Resolved Fluorescence Imaging of Androgen Receptor and Prostate-Specific Antigen in Prostate Tissue Sections. J Histochem Cytochem.

[20] Wu L, Liu Q, Ruan X, Luan X, Zhong Y, Liu J, Yan J, Li X (2023;Jun 29). Multiple Omics Analysis of the Role of RBM10 Gene Instability in Immune Regulation and Drug Sensitivity in Patients with Lung Adenocarcinoma (LUAD). Biomedicines.

[21] Pértega G N, Vizcaíno J R, Gouveia C, Jerónimo C, Henrique R M, Lopes C, Baltazar F (2013;May). Monocarboxylate transporter 2 (MCT2) as putative biomarker in prostate cancer. Prostate.

[22] Stewart J, Fleshner N, Cole H, Sweet J (2007;Jul). Comparison of annexin II, p63 and α-methylacyl-CoA racemase immunoreactivity in prostatic tissue: A tissue microarray study. J Clin Pathol.

[23] Wu L, Zheng Y, Ruan X, Wu D, Xu P, Liu J, Wu D, Li X (2022;Jan 1). Long-chain noncoding ribonucleic acids affect the survival and prognosis of patients with esophageal adenocarcinoma through the autophagy pathway: Construction of a prognostic model. Anticancer Drugs.

[24] Hameed O, Sublett J, Humphrey P A (2005;May). Immunohistochemical Stains for p63 and α-Methylacyl-CoA Racemase, Versus a Cocktail Comprising Both, in the Diagnosis of Prostatic Carcinoma: A Comparison of the Immunohistochemical Staining of 430 Foci in Radical Prostatectomy and Needle Biopsy Tissues. Am J Surg Pathol.

[25] Kristiansen G, Fritzsche F R, Wassermann K, Jäger C, Tölle A, Lein M, Stephan C, Jung K, Pilarsky C, Dietel M, Moch H (2008;Sep 16). GOLPH2 protein expression as a novel tissue biomarker for prostate cancer: Implications for tissue-based diagnostics. Br J Cancer.

[26] Wu L, Zhong Y, Yu X, Wu D, Xu P, Lv L, Ruan X, Liu Q, Feng Y, Liu J, Li X (2022;Oct 1). Selective poly adenylation predicts the efficacy of immunotherapy in patients with lung adenocarcinoma by multiple omics research. Anticancer Drugs.

[27] Lindner V, Waydelich A, Chen C C, Jones C, Stratton S P (2021;Aug). Performance comparison of anti-p504s (SP116) Rabbit Monoclonal Primary Antibody vs. Monoclonal Rabbit Anti-Human AMACR clone 13H4 when duplexed with VENTANA Basal Cell Cocktail (34βE12+p63) as a diagnostic aid for prostatic adenocarcinoma using immuno-histochemistry. Virchows Arch.

[28] Farinola M A, Epstein J I (2004;Oct). Utility of immunohistochemistry for α-methylacyl-CoA racemase in distinguishing atrophic prostate cancer from benign atrophy. Hum Pathol.

[29] Daoud N A, Li G, Evans A J, van der Kwast T H (2012;May). The value of triple antibody (34βE12 + p63 + AMACR) cocktail stain in radical prostatectomy specimens with crushed surgical margins. J Clin Pathol.

[30] Sircar K, Gaboury L, Ouadi L, Mecteau M, Scarlata E, Saad F, Aprikian A, Tanguay S, Lapointe S, Lussier C, Miletti T, Lanoix J (2006;Jul 15). Isolation of Human Prostatic Epithelial Plasma Membranes for Proteomics Using Mirror Image Tissue Banking of Radical Prostatectomy Specimens. Clin Cancer Res.

